# Artificial intelligence and real decisions: predictive systems and generative AI vs. emotive-cognitive legal deliberations

**DOI:** 10.3389/fsoc.2024.1417766

**Published:** 2024-10-30

**Authors:** Francesco Contini, Alessandra Minissale, Stina Bergman Blix

**Affiliations:** ^1^Institute of Legal Informatics and Judicial Systems, National Research Council, Bologna, Italy; ^2^Department of Sociology, Uppsala University, Uppsala, Sweden

**Keywords:** emotions, empathy, legal decision making, predictive justice, generative AI

## Abstract

The use of artificial intelligence in law represents one of the biggest challenges across different legal systems. Supporters of predictive systems believe that decisionmaking could be more efficient, consistent and predictable by using AI. European legislation and legal scholars, however, identify areas where AI developments are at high risk or too dangerous to be used in judicial proceedings. In this article, we contribute to this debate by problematizing predictive systems based on previous judgments and the growing use of Generative AI in judicial proceedings. Through illustrations from real criminal cases in Italian courts and prosecution offices, we show misalignments between the functions of AI systems and the essential features of legal decision-making and identify possible legitimate usages. We argue that current predictive systems and Generative AI crunch the complexity of judicial proceedings, the dynamics of fact-finding and legal encoding. They reduce the delivery of justice to statistical connections between data or metadata, cutting off the emotive-cognitive process that lies at the core of legal decision-making.

## 1 Introduction

Digital technologies have contributed to handling legal proceedings for more than 30 years. Initially through case registrations and case management, later with fully-fledged e-justice platforms, they provided the digital workplace needed to run judicial proceedings from filing to disposition. The first wave of technological deployment mainly concerned procedures, records, case files and the collection of judgments in dedicated databases.

In the last decade, artificial intelligence triggered a second wave of innovation. The promise of robot judges and systems predicting judicial decisions caused the excitement of many (Ashley, 2017; Chen, 2019). However, the first systems applied in real settings generated bias, discrimination against minorities, and undue and potentially dangerous pressures on decision makers (Angwin et al., 2016; Morison and Harkens, 2019; Morison and McInerney, 2024). Over the years, the rise of issues and ethical concerns about AI in several fields cooled down the enthusiasm and hype on automatic and robotic judicial decisions. As a result, several ethical codes have been approved (Lupo, 2022) and, more recently, the European Union passed the AI Act.^1^ In this article, we contribute to the debate on the role of AI in judicial decision-making by problematizing the use of predictive systems based on natural language processing of previous judgments and of generative AI (GenAI) based on large language models. We draw on illustrations from Italian data collected in the Justemotions project consisting of observations of deliberations and interviews with magistrates showing the emotive-cognitive dynamics of real decision-making. This unique data set is used to reason on the implications of introducing predictive and generative AI systems in judicial and prosecutorial decision-making, highlighting the importance of accurately accounting for how human interpretation works in real legal practice. We argue that both predictive justice systems and GenAI, in their distinct forms, introduce logical simplifications that crunch the complexity of judicial proceedings and alter the dynamics of fact-finding and legal encoding. These technologies cut off the emotive-cognitive process of legal decision-making, reducing the delivery of justice to statistical connections between data, metadata or text. The following sections describe the features and logic of predictive systems and GenAI; provide a brief explanation of the methods used to collect data and of the characteristics of the Italian criminal procedure that are relevant to understand our illustrations; and compare real deliberations to AI, highlighting the integration of emotional dynamics to fact-finding and interpretation. In the conclusion, we discuss the implications of our empirical findings and identify possible risks and opportunities.

## 2 AI in justice systems

AI entered into court operation mainly through systems supporting text processing (Reiling, 2020), in the form of *speech-to-text* and *anonymization of judgments*. For years, speech-to-text or language editing have been based on AI-systems embedded in everyday word processing applications. Today, professionals involved in judicial proceedings use these systems to write (dictate) and check the language. Speech-to-text improves writing speed, making it possible for judges/clerks to write minutes during hearings. The second type of systems—those anonymizing judgments—are designed to allow the publication of judgments compliant with privacy regulations. AI based anonymization erases personal data from judicial decisions, with huge time saving. The outputs of both these systems can be easily checked by users, and are not considered by the European AI Act. In contrast, direct usages of AI in legal processes, particularly applications influencing judicial deliberation, are acknowledged by the EU AI Act as “high risk” (Chapter 2 AI Act).

Criticisms of these systems touch upon various arguments, including systems’ bias, limited accountability (Chiao, 2019; Gualdi and Cordella, 2021), complexity and lack of understandability of AI and consequently of justice administration (Re and Solow-Niederman, 2019). Lack of explanations about the machines’ suggestions (Mittelstadt et al., 2016) can result in undue influence on the judicial function (Contini, 2024). Further critiques stress the black-box problem (Bathaee, 2018), magnified when private companies own these systems, which are non-accessible to third-parties, and the risk of jurisprudential ossification due to the *effect mouton* (all judges follow uncritically the decision suggested by the machine) (Garapon and Lassègue, 2021).

The predictive systems in use, or more often under development, fall into various categories: those estimating the recidivism risk not further considered in this article,^2^ and those supporting sentencing (Bagaric and Hunter, 2022) or designed to predict and/or suggest a decision by identifying a case (or cases) very similar or identical to the one to be decided, through statistical analyses and probabilistic calculations. These systems are designed to exclusively fulfill the specific function of predicting and/or suggesting the judicial decision. In contrast, GenAI has multi-purpose functions not established in advance. They intend to interact with users through questions and answers and are autonomous in generating text (but also other outputs like images or sounds) in reply to prompts. For this reason, these applications are also referred to as general-purpose AI systems in the EU AI Act. Answer and text generation is probabilistic, based on statistical relationships discovered during training processes (Ferrara, 2024).

### 2.1 Predictive systems

Predictive justice systems allow forecasting possible outcomes of disputes based on previous solutions to analogous or similar cases. They entail a broad spectrum of applications mainly (even if not exclusively) based on supervised machine learning (Galli and Sartor, 2023, p. 173) through which data sets first are annotated and then algorithms are trained and supervised to predict outcomes and recognize patterns. Predictive systems are classified as high risk by the EU AI act (Annex III Art-8). A typical example of how predictive systems work is the approach developed by Aletras et al. (2016, pp. 3–19), Medvedeva and McBride (2023) and Collenette et al. (2023) to predict decisions of the European Court of Human Rights (ECtHR) dealing with articles 3, 6, and 8 of the Human Rights Convention. The authors state that the system is designed to “rapidly identify cases and extract patterns that correlate with certain outcomes” (pp. 3/19). The algorithm, using natural language processing and machine learning, predicts whether the Court will rule a violation of a specific provision of the European Convention on Human Rights (ECHR) with a 79% of accuracy. The tool works on information from previous judgments available in the online database of the ECtHR. The logic behind the system is that when uploading a new petition (“application” in ECtHR jargon), the system checks the similarities with previous cases and predicts the decision of the Court. The checking between the “new petition” and the existing body of judgments is made automatically. In all the cases of the ECtHR, predictions assume that there is enough similarity between specific chunks of the text of published judgments and complaints lodged to the Court.

Discussing Aletras and colleagues’ work, Reiling (2020), noticed that AI algorithms in this system do not work on the entirety of texts generated from previous cases. Initially, the system singles out judgments included in the ECtHR online database, in which cases classified as inadmissible requests are not available. In the next step, judgments are tagged through semantic annotations, associating chunks of the texts (sentences, words) with concepts. As a result, each judgment is classified based on several variables: procedure, circumstances, facts, and relevant law. These annotations transform the unstructured text^3^ of each judgment and its flow of arguments into structured data suitable for statistical elaboration based on AI techniques. In this system, annotations can be made by humans, automatically by a machine, or by a mix of the two.

The two steps are examples of functional simplifications on two levels. First, judicial cases are reduced to final judgments,^4^ whereas other documents in the case files and the dynamics that may affect the unfolding of proceedings, such as preliminary hearings, trials, and courts’ deliberations are cut off. Second, arguments and nuances of judgments are streamlined into machine-processable concepts (annotations and learning algorithms).

In similarity to Aletras and colleagues’ system, other predictive systems already in use or under development have in common the classification of existing procedural documents through tags or semantic annotations. Differences entail mainly the ways in which users interact with the data generated by the systems to get predictions. Some systems are designed to allow users to query the judgment database through a pre-established subject matters list, following decisional trees. This is the case of the System for predictive justice of the Court of Appeal of Brescia^5^ for labor and company law. Once the thematic area of interest has been selected (either labor or company law), the system provides pre-established pathways to identify a case, either identical, or similar to the one searched for by users. This operation is referred to as predicting the *sought-after solution*. Other systems allow queries in natural language (i.e., the common language, usually juxtaposed to queries based on Boolean or other non-necessarily intuitive criteria). This is the ambition of the system experimented by the Court of Appeal of Venice (Musella, 2023), the Tribunal of Pisa (Nencini, 2024)^6^ and several commercial services promising to provide the most relevant answers to complex legal questions by database searches. These simple search methods identify, among the vast jurisprudence available on the platform, the judgments that better fit with the query^7^. If a case with the same features has been already decided, that judgment(s) identified by the machine will predict the decision. Hence, the prediction is based on similarities between the case and the existing jurisprudence. These systems receive high regards within the judicial community even in civil law countries like Italy or France, where the *stare decisis* (i.e., following the precedent) principle does not apply. They transform the content of a judgment into fragments that can be elaborated through machine learning and other AI techniques.

To a minor extent, predictive systems also aim to address prosecutorial decision-making. In 2021, a group of Chinese researchers claimed to have created the world’s first AI prosecutor (Petersen, 2022). The robot, tested in the Shanghai Pudong People Procuratorate, was set to press charges based on 1,000 “traits” from the human-generated case description texts. The AI prosecutor was “trained” using 17,000 real life cases from 2015 to 2020 and was considered able to identify and press charges for the eight most common crimes in Shanghai with 97% accuracy.

In sum, the philosophy behind all these systems is that if the law is objective, repeatable and based on predetermined and binding rules, its application can be foreseen, combining “big data” analysis and “machine learning” techniques (Medvedeva and McBride, 2023). Hence, these models reproduce judicial reasoning through syllogistic logic and work on pieces of “knowledge” mainly extracted by judgments.

### 2.2 Generative AI

GenAI systems like ChatGPT, CoPilot or Gemini, are a new family of applications increasingly used in judicial proceedings (Pierce and Goutos, 2024; Grossman et al., 2023b). They are based on Large Language Models that, through probabilistic calculations, predict the next word in a sentence. Chatbots with GenAI reply to ‘prompts’, i.e., natural language instructions given to the system, to obtain an output based on pre-trained data sets (Courts of New Zealand, 2023, p. 1), hence are multipurpose. In legal work, they can be asked to summarize documents, select facts from different stories of an event as collected in interviews, or look for similarities and differences between stories. Users could also ask to separate the issues disputed from those agreed upon and check prosecutors’ arguments against those of the defense. Finally, a judge could ask the GenAI system how to decide a case. In contrast to the systems discussed earlier, GenAI can be used privately and without external control and is freely accessible on the Internet (the more advanced versions for a subscription fee).

Before venturing into the analyses of GenAI in judicial proceedings,^8^ it is necessary to explore its actual usage and define uses that are considered acceptable. The suspicion that judicial officers took advantage of these systems in the privacy of their chambers proved well founded when some of them began to report the use of GenAI into judgments. Evidence is anecdotal but constantly growing. The first known case (February 2023) is by a Colombian judge who asked a GenAI system to help him decide a case involving the medical insurance of an autistic child. The dialogue (question and answer) between the judge and the bot was reported in the judgment and sparked a debate (Gutiérrez, 2024). The following month, an Indian judge asked ChatGPT for advice about granting bail to a murder suspect (Grossman et al., 2023b). At the same time, a Pakistani judge made an ‘experimental’ usage of ChatGPT to rule a sexual assault case. The judge asked for a legal definition of the concept of “consent” and included the response in the judgment (Web Desk, 2023). In September 2023, an English appeal judge admitted having used ChatGPT to summarize an area of law in which he was an expert. He received an answer that he felt was acceptable and included it in the judgment (Farah, 2023). More recently, a Dutch judge was criticized for having asked ChatGPT to figure out the ‘current average price of electricity’, as well as the ‘average lifespan of solar panels’, to calculate damages in a case (Amalaraj, 2024).

These different examples became public because the judges referred to using GenAI in various ways. They show that judges can use such systems unofficially and without previous approvals or checks. There are cases indicating that other legal professionals, such as lawyers and prosecutors also use GenAI in this informal and undisclosed way (Grossman et al., 2023a). Furthermore, they show the multipurpose usage of GenAI. Functions can range from asking the definition of a legal concept (Pakistan) to summarizing a legal area (England), from exploring the conditions for granting bail (India) to going straight to the point and checking how the case should be adjudicated (Colombia).

As a result of these episodes testifying an exploratory use of chatbots, several bodies issued guidelines to regulate their use (Contini, 2024, p. 11–16). In December 2023, the Courts and Tribunal Judiciary of England and Wales released of the first specific guidance to address the use of GenAI in judicial proceedings (Courts and Tribunals Judiciary, 2023). The document highlights many limitations and risks of GenAI and suggests possible usages. The guidelines make clear that any information entered into a public AI chatbot is made publicly available worldwide. Hence, using confidential information in a chat with a GenAI represents an inappropriate disclosure. The document further highlights that GenAI systems are prone to errors. They can make up fictitious cases, citations or quotes, or refer to legislation, articles or legal texts that do not exist. In this way, they can provide incorrect or misleading legal information or make factual errors. Since GenAI responses—as any other AI based system—are based on the dataset they are trained upon, they will reflect errors and biases in training data. Moreover, in the legal field, it is often difficult and sometimes impossible to understand if the answer is based on the US, UK or other jurisdictions. Despite these serious limitations, the Courts and Tribunal Judiciary of England and Wales guidelines identify possible usages of GenAI limited to summarizing texts, conditioned to verifying the summary’s accuracy, and to side activities like getting “suggestions for topics to cover” or drafting emails and memoranda. In said guidelines, GenAI is not recommended for legal research analyses or other case-related activities. Furthermore, the use of GenAI must not necessarily be disclosed. Judicial officers are personally responsible for their writing, particularly those forming the case files. Judges are not generally obliged to describe the research or preparatory work leading to the final judgment. The same applies for legal representatives, which “are responsible for the material they put before the court/tribunal and have a professional obligation to ensure it is accurate and appropriate. Provided AI is used responsibly, there is no reason why a legal representative ought to refer to its use” (Courts and Tribunals Judiciary, 2023, p. 5). In this article, we draw on the possible usages identified by the Courts of England and Wales, to consider GenAI implications for summarizing case related documents.

## 3 Methods and research context

This article uses Italian data collected within the Justemotions project financed by the European Research Council (757625). The project investigates, using ethnographic methods, the emotive-cognitive process of legal decision-making in courts and prosecutors’ offices in Italy, Sweden, the US, and Scotland. In Italy, we followed cases of fraud, intimate partner violence (IPV), homicide, rape, theft, and libel, totaling 80 criminal cases. We shadowed and interviewed 34 prosecutors and 40 judges, observed 158 hearings and 47 deliberations (40 at tribunals and seven at the court of appeal).

During shadowing (Czarniawska, 2008), we followed legal professionals during their workday, and engaged in reflection on their activities and the development of their decision-making. In observations of trials, we focused on legal professionals’ presiding in hearings and examining witnesses and defendants and on their emotional expressions. During deliberations, we were attentive to the interaction between judges and to the reasoning leading to the final verdict. We also used pre-hearing and post-hearing semi-structured interviews, to add participants’ own reflections about each case, their decisions and emotions. Lastly, we analyzed written judgments, to understand how the reasoning occurred during the deliberation was then transformed into a legal story.

In this article, we use examples from different types of emotional dynamics that we analyze elsewhere in a more comprehensive manner (Bergman Blix and Minissale, 2022; Törnqvist and Wettergren, 2023; Minissale, 2024; Bergman Blix and Törnqvist, 2024; Bergman Blix, 2019). Since the scope of the current article is to contribute to the debate on the risks and opportunities underpinning the use of AI in legal decision-making, we use the Justemotions data to explore misalignments between real decision-making, on the one hand, and predictive justice and GenAI, on the other hand.

In our examples, we meet judges and prosecutors, whose names are fictitious and experience indicted by an +five age range, dealing with criminal trials at different stages of the criminal process, from preliminary investigation to deliberation. In Italy, criminal proceedings start with an investigation conducted by the public prosecutors’ office. Triggered by a police report or a complaint, the prosecutor directs investigative police to examine the crime scene, interview witnesses, and gather evidence. At the end of the investigation, the prosecutor can dismiss the case or issue the indictment, which outlines the charges and the evidence gathered during the investigation. The subsequent phase is a preliminary hearing during which a judge reviews the evidence. If the case is not dismissed, the judge decides the next steps after considering the parties’ requests.

The trial is an adversarial process where prosecutor and defense present their case before a judge, a panel of three judges, or a special panel composed of two judges and a jury of six laypersons. The parties can appeal the first instance court’s decision at the Court of Appeal, which reviews cases considering evidence and legal matters. Both defense lawyers and prosecutors can ask the Court of Cassation to review the decisions taken at the appeal level. The Cassation considers only legal issues.

Three fundamental legal principles shape the criminal procedure: its adversarial structure (*contraddittorio*), orality (*oralità*) and immediacy (*immediatezza*). According to the adversarial principle, prosecutor, defense and eventually the victim’s counsel can present their evidence, examine and cross-examine witnesses, challenge mutual arguments and argue their case before an impartial judge. The principle of orality emphasizes the importance of the oral presentation of evidence during the trial allowing the judge to hear witnesses’ and parties’ statements directly, creating a dynamic and interactive trial. The immediacy principle entails that the judge must have direct experience of the evidence and depositions presented during the trial, observe the demeanor of those involved in the procedure, assess witnesses’ credibility, and make decisions based on first-hand knowledge acquired during the trial. This principle minimizes reliance on written records and enhances judges’ ability to evaluate the evidence in real-time. Taken together, these principles shape procedures and hearings and mold the context in which evidence is built and assessed and objectivity is constructed.

## 4 Contrasting real decision-making with AI systems

Legal decision-making is a process requiring fact finding, fact interpretation, and legal encoding—the translation of lay stories into legal stories purified of their subjective elements (Bergman Blix and Minissale, 2022). This section shows that legal professionals evaluate cases in small steps, fragmenting the story in separate pieces, interpreting those pieces both separately and in relation to one another. Legal professionals reduce and simplify the case story to selected events relevant from a legal perspective (i.e., legal check), but also need to verify that the constructed legal narrative holds on social reality (i.e., reality check). The gradual simplification of the case story, accompanied by the reality and legal checks, build on cognitive and emotional processes, such as empathic attuning, interest in relevant issues as well as disinterest in irrelevant aspects (Bergman Blix and Minissale, 2022). These emotional dynamics are important to arrive at a judgment that accounts for the specificities of each case. Predictive systems based on previous judgments, instead, work with already simplified versions of the facts at stake in a legal dispute, where judgments are annotated and connected by machine learning algorithms, purifying stories from their nuances and details. Even if through different statistical systems—such as LLM predicting the likelihood of the word coming next based on training data—information loss also occurs in summaries made by GenAI.

### 4.1 Deliberation as an emotional reflexive dialogue with jurisprudence

The following example is a case of theft with six individuals accused of stealing mimosa flowers from a private garden. From the fieldnotes taken while observing the deliberation, it is possible to notice how Tribunal Judge Ines (40+) fragments the story to establish whether the theft is limited to an attempt, and if it there is the aggravating circumstance of “violence against things.” The judge critically reflects on previous rulings of the Court of Cassation about seemingly similar cases. This allows us to see the effort made by the judge to identify nuances in cases that are similar in the big picture but different on a closer look. That is, the effort made by the judge is not just to frame the case in the big picture but to discover and account for the details that qualify the story from a factual and legal perspective.

*Judge Ines: “Okay, we have several people accused of stealing mimosas in a private home on women’s day […] the police watched them all the time as they took the flowers” […] Judge Ines re-reads the police report out loud […] She circles in the report “The tree had split-up and broken branches; there was a clear degree of damage to the tree.” “So, there is also damage.” Keeps browsing and says: “I would say that there is really nothing to do.” Ines remains silent and then reads the defense brief: “They do not take possession, according to the defense.” Ines searches on her computer and finds a judgment about a case similar to the present one, where a person took some objects from another car and put them inside his car. In this case, the Court of Cassation said that it was an attempted theft. “Just like in a supermarket theft, the security guards watched them all the time. It is necessary to understand if there is an attempt. However, there is violence because—says the Court of Cassation—there is violence even when you steal fruit from a tree—lemons, for example—because if you do not collect them in a certain way, you cause some damage.” Ines searches for further jurisprudence on attempt on her computer. “So, in 2018, the Court of Cassation says that the theft is in the consummated form when the defendant maintains, even if for a short time, the full and autonomous availability of the stolen goods. So, for us, too, it is theft, because they had branches in the car. In my opinion, the first ruling of the Court of Cassation relates to a partially different hypothesis, because here the police only saw part of the action, they saw a part of the theft but there were already branches in the car when they arrived. This is different from the hypothesis in which the police observe the theft in a supermarket from the beginning.”*

In this excerpt from the deliberation, the judge’s reasoning fluctuates between the evaluation of the legal categories of “violence against things,” “attempted theft,” and “theft.” Her reasoning follows a complex journey in which the construction of a coherent legal story is preceded by a more or less chaotic navigation through the story at stake. Early on during the deliberation, Ines seems to feel certainty about the final decision (“I would say that there is really nothing to do”) because “the police watched them all the time as they took the flowers” (i.e., theft) and “[t]he tree had split-up and broken branches; there was a clear degree of damage to the tree” (i.e., violence). The judge, however, uses doubt to resist her certainty (Minissale and Bergman Blix, 2024) and dig deeper into the case. She re-reads the defense brief and analyzes previous rulings of the Court of Cassation. A first ruling seems to be in favor of the “attempt” hypothesis, but Ines detects a crucial difference between the cases, as in the current one the police observed only part of the theft *in vivo*. To reinforce her certainty about this line of reasoning, Ines searches for more jurisprudence and compares specific factual elements of the different stories under consideration. Reading the defense brief and previous rulings prompts the judge to reflect and find connections, patterns and ultimately making sense of the case to reach a decision. She constructs a legal story that considers the versions of both parties (adversarial principle) and is coherent with the reality under scrutiny. In the quest for certainty about the final decision, the reality check and legal check are intertwined.

Seen from a distance, all trivial thefts might look alike, but as the mimosa case demonstrates, facts can be unclear also in this type of cases. It is only by digging into the small details that relevant differences between prior judgments and current cases emerge. The structural features of predictive justice exclude those details. Summaries made by GenAI building on case briefs or judgments would incentivize shortcuts and a summary consideration of legal and factual details.

The richness of the full case file is not considered because the system works on statistical calculations of the annotations and their connections made on a written judgment or on selections of relevant points made by GenAI. In our example, the judge critically examines facts and previous jurisprudence about similar cases after considering the different qualifications of the events presented by the defense. The trial dynamics, its adversarial and oral structure as mentioned earlier, are designed precisely to share different understandings and qualifications of the facts at stake, to give the judge the information required to reach a decision.^9^ Facts become progressively clear, while their selection and qualification for the final judgment is built in interaction and dialogue with the legal framework and the jurisprudence of the Court of Cassation. A reflexive dialogue between the judge and the jurisprudential archive is required to explore and define factual and legal issues (Giabardo, 2023). Here emotions, particularly epistemic feelings of doubt, uncertainty, interest, curiosity, and empathy, are key to maintain “sensitivity to the situations” (Gaboriau, 2018) and to prompt a reflexive problematization of knowledge and information (Bergman Blix and Minissale, 2022; Törnqvist and Wettergren, 2023; Minissale, 2024). This emotional-reflexive dialogue (Burkitt, 2019), however, is not considered or rather removed in the logic of the predictive systems, where the goal is to suggest the decision based on previous judgments as identified by machine learning processing of historical case data. In this case, the GenAI reduction of data and streamlined analysis (i.e., tagging) would not allow judge Ines’ back-and-forth reflections on different versions of the facts of the case in dialogue with previous judgments. Nor would it instigate the epistemic emotions of interest, doubt and eventually settled certainty that guide the deliberative process, making it possible to balance legal and reality checks to reach a sound judgment.

Another example where we can see the importance of the reality check together with empathic interpretation of the facts at stake, is the following case of IPV and sexual violence decided by a panel of three judges at the tribunal. During the deliberation, Judges Enrico (Head of the Panel, 55+), Beatrice (45+), and Sonia (honorary judge, 45+) evaluate the victim’s credibility by trying to make sense of the relationship between the couple (victim and defendant). They engage in joint *empathic attuning* (Bergman Blix, 2019) to understand the victim’s perspective and the defendant’s personality, alternating this with the legal check (i.e., evaluation of the story under the legal framework). By contrasting fieldnotes from the deliberation with the final written judgment, we show how the empathic reasoning used to understand the facts at stake disappears in the final text. Simplification is embedded in judicial procedures and occurs at different levels as procedural events and hearings are reduced into text from the first instance to the appeal. We stress that the additional simplification brought in by predictive systems and GenAI becomes an obstacle to considering details of the story that open up for emphatic imagination and attuning relevant for its legal categorization. In the extract below we see how the interpretation of facts described in legal transcripts gives rise to empathic reasoning necessary to assess what goes on in a case:

*Enrico (looking at Beatrice): [The victim] talked about the sexual violence in a particular way. The defendant was stunned. If I took my notes correctly, she went into [one of the witnesses] car, with her handbag, she put her handbag in the backseat, [the defendant] attacked her physically, with his body, picked up her handbag, somehow convincing her to get into his car again.*

*Beatrice: everything in a great agitation…*

*Enrico: a very particular sexual violence…*

*Beatrice: a person with whom she had a relationship…*

*Enrico: that is…he did not bring me into the forest and held me there for an hour, raping me…but it is part of that context…*

*Beatrice: also, because she talked about particular sexual requests. Consistent with his sexuality…*

*Enrico: as the civil part said, it was a gesture of affront…*

*Beatrice: done in a public space…*

*Enrico: it is part of his way of conceiving the relationship, sex…a bit like witness told us… it is not that he wanted to steal the handbag, but for a sentimental reason, so to speak [he took the handbag]. So, in short, he reacts like that because he wanted to deal, from his point of view…*

*Beatrice: in his own way, he wanted to resume his position…he substantially had not worked out the separation from her….*

*Enrico: let us say not worked out AT ALL!*

*Beatrice: The only thing going against the victim’s credibility would be that she did not report it immediately?*

*Enrico: well, not very immediately…but when she returns a bit calm, she recovers, in that moment she tells a full story of what happened, and in this story, there is also the moment of the finger…*

*Beatrice: she appears reliable overall…when a fact happened only with two people there, the only thing is that of credibility…*

*Enrico: her narration was short but precise…surely, when she was heard […]*

*Beatrice: she does not dwell on superficial things during her examination, neither she tries to exaggerate facts…which have been confirmed…*

To decide on the victim’s credibility—whose word is enough for conviction in this type of crime—the judges in unison analyze the sequence of actions allegedly done by the defendant in a step-by-step fashion. Emphasis is placed both on demarcating legally relevant facts in the victim’s narrative (“she went into the witness’ car, with her handbag”), and on understanding the nuances of the story. Reflections on the defendant’s “sexuality,” “a gesture of affront,” “his way of conceiving the relationship,” “he wanted to resume his position,” “he had not worked out the separation from her…” all together render visible how the judges collectively use empathy to interpret the relationship between victim and defendant. This practice is not covered by the legal method, but is nevertheless crucial to evaluate the credibility of conflicting stories. They empathically immerse themselves in the victim’s story indicating an abusive relationship (“sexual violence,” “a person with whom she had a relationship”), even describing it in a first-person account (“he did not bring *me* into the forest and held *me* there for an hour, raping *me*”) to relive the story from the victim’s perspective. They also engage in a fleeting superficial empathic attuning with the defendant depicting his dominant role in his relationship with the victim (“was stunned,” “…consistent with his sexuality”). In this effort, the details of the case, the personal experiences of the trial (immediacy principle), and the richness of verbal and non-verbal communications emerge through judges’ memory of the hearings and personal notes taken from the bench. This reality check comes out as necessary to establish whether the alleged episode of sexual violence—as told by the victim—could have actually occurred. When, instead, the panel describes the narration of the victim as “short but precise,” emphasizing that “she does not dwell on superficial things during her examination, neither she tries to exaggerate facts…which have been confirmed…,” their attention goes back to the legal check—what is legally relevant to establish credibility based on the criteria defined in the jurisprudence. In this example, we see that when relevant *facts* are established, they require interpretation to fit within legal categories (credibility). Interpretation builds on a thorough assessment of human relations and emotions, which are dimensions removed from the logic of predictive systems and GenAI working on cold statistical elaboration based on textual analyses (Galli and Sartor, 2023; Contini, 2024). The judges in the appellate court also analyze text (rather than oral evidence), but their analysis, relies on joint empathic attuning with descriptions of facts offered by witnesses, victims and their legal representatives. Through reflexive-interpretative work, relevant facts become progressively clear and can be legally encoded. Notably, the final judgment does not reveal these reality checks based on joint empathic attuning performed by the judges:

*On the basis of the evidence, it is believed that the criminal liability of the accused should be affirmed for all the charges. Underlying the affirmation of the defendant’s responsibility there are, first of all, the accusatory statements made by the victim, which appeared to be fully credible. […] In this regard, it is observed that the narrative of the victim appears to be consistent in the essential points of the events. There are no expressions of animosity or rancor towards the accused that would lead one to believe that the facts narrated did not take place, that the victim narrated them in a deliberately more serious manner, or that she is animated by a slanderous intent. The circumstances told by the victim are confirmed by multiple and timely corroborations, in particular: by the statements of witnesses 1, 2, 3; by the content of the e-mails produced […] by the medical certification acquired in the files […] by the content of the police record.*

When comparing the reasoning during the deliberation with the final judgment, we see how the legal method and writing style cut off the reality check and the emotive-cognitive processes behind the final verdict, such as the joint empathic attuning by the three judges during the deliberation. These “hidden” dynamics refer to important temporal and relational dimensions of legal decision-making, where evaluations are made in small steps, fragmenting the narrative, considering the nuances of the case, which is necessary to avoid simplifications based on previous cases or brief summaries, aligning legal narratives to social reality.

In sum, predictive systems work with annotations based on fragments of texts that are derived from abstract legal categories, such as linearity, coherence, lack of contradictions, and restrained declarations for evaluating credibility (Collenette et al., 2023). As depicted in this IPV and rape case, these abstract categories require interpretation linked to the specificities of each individual case. The interpretative work demands empathic attuning into the different stories at play. However, in the final judgment, the traces of this vital part of the process remains hidden. GenAI summaries cannot be used for these purposes since they minimize the information required for empathic attuning.

### 4.2 The necessity of emotional-interactional information

The reality check described in the previous section returns in the following examples in a slightly different form, as it refers to legal professionals’ need to incorporate emotional-interactional information about the person giving testimony and their storytelling in diverse types of texts, such as police reports, transcriptions of witnesses’ declarations, and minutes of the hearing. We argue that this type of information is crucial to include in, and account for, in analyses made by predictive systems and GenAI. Furthermore, even when this type of information is present in the text and can thus be potentially tagged and processed by predictive systems and GenAI, it requires human interpretation to validate a meaningful understanding of the case. In the following example, we show how emotional-interactional information is used by prosecutors and judges in their decision-making practices.

During an interview, Prosecutor Stefano (40+) recounts a case of IPV where the details in the police report indicated a serious offence. Before taking any decision, however, Stefano decided to personally hear the victim as he could not find sufficient elements to categorize the type of criminal behavior.

*Stefano: [the police] called me around 3 a.m. saying they had intervened inside a house a couple […]. And the woman recounted to the police that she was arguing with her husband about a situation that was festering, and in the course of the argument the man took their little daughter in his arms, lifted her up, SHAKED HER and while doing so he THREATENED his wife. So, he does not threaten to harm the child, but it is a gesture that is objectively ambiguous, equivocal, even towards the child. In the course of this quarrel, when the police intervene, there is no remaining evidence of the crime. The lady had no signs of injury, he had pulled her hair, he had slapped her and left no marks. And the house was not in particular disorder. And so, I, that very day…when they brought me the complaint of the lady, I see that it is badly done, there are not many elements. So, I ordered her to be brought to me [for a personal examination]*

*Interviewer: bad from what point of view?*

*Stefano: Technical. I cannot reconstruct the story of this couple from that report, nor can I understand if there is actually abuse, and above all this fact of the little girl, I cannot understand it. So, I had the lady brought immediately to me, in the afternoon.*

In the quote, Stefano draws attention to missing aspects in the account presented by the police, which he considers important to make sense of a potentially grave criminal action (shaking the child). He highlights the need of “reconstructing the story of this couple” in order to decide on precautionary measures and hopes to solve this doubt through a direct interaction with the victim. In the continuation of the interview, Stefano describes how the interaction with the victim enhanced his understanding of the social context underlying the specific episode described in the police report:

*Stefano: I heard her and, in the evening, I wrote the request for precautionary measure, a restraining order (‘prohibition to approach her’). Because, actually, this fact that seemed bad, the lady actually tells me well, in detail, about her life with this man. So, she was here, at my place. A simple person. […]. She describes a story, which is certainly a story of IPV and of a relationship that no longer works and from which she wants to free herself, but… basically… he does not drink, he does not use drugs, it wasn’t a bad story. It was a story of marginalization, of poverty, of a family where he was constantly obsessed with not being able to cope financially. And there were a series of quarrels that, no matter how hard they both worked…it was a family relationship that NO LONGER WORKED and that HE, as a male, wanted to solve in an arrogant and violent way. So, it was a BROKEN, DEGENERATED family situation, but there was no proven pattern of violence.*

The most interesting part of this quote comes in the final remark on the lack of a “proven pattern of violence.” This signifies the missing proof regarding the “habitualness” of the conduct, which in the Italian legislation is a prerequisite for the crime of IPV to exist. From the police report, the story was originally interpreted as indicative of a serious offence—IPV—(“this fact that seemed bad”), but is reframed as one with a lower criminal disvalue (“it wasn’t a bad story”), and most importantly as one missing the requirement of ‘pattern of violence’. In order to solve his feeling of doubt and settle on a decision that this is not a case of IPV, prosecutor Stefano needed to put the specific events into their social context, and empathically attune to the perspectives of both parties. Reconstructing the nuances of the story at stake through a direct interaction with the victim generates in the prosecutor a clearer understanding of the events than in the police report. The social context both clarifies that the defendant neither abuses alcohol or drugs, and that the family lives under severe marginalization and poverty leading to constant conflict. Empathically attuning with the victim, prosecutor Stefano acknowledges her fear of the defendant’s “arrogant and violent way” and want to “free herself,” leading him to ordering a restraining order. Empathically attuning with the defendant, instead, Stefano acknowledges his struggle with poverty, causing aggressive, but not legally abusive behavior. Taking in both sides, Stefano assesses the case as a “BROKEN, DEGENERATED family situation,” without “proven pattern of violence.”

Textual descriptions of cases are those used by AI systems meant to aid or substitute prosecutors, like in the Chinese example mentioned earlier. Predictive systems base their predictions on previous judgments/indictments, hence on documents providing a key, but radically simplified exposition of facts, legal issues and their connections. Information regarding emotions, non-verbal behaviors and the nuances of the case can be crucial to take decisions from the investigative phase, as visible in prosecutor Stefano’s example. In our material, prosecutors often stressed the importance of emotional-interactional elements in order to evaluate witnesses’ and victims’ credibility. Prosecutor Anna (30+), for instance, clarifies that the benefit of a direct perception of the victim’s narration is being able to “see their expressions, their gestures, their reactions,” which enhances one’s certainty about perceived credibility. In real life, prosecutors rarely have the time to personally hear the complainant due to the high number of investigations (especially on IPV allegations) that they handle. As a consequence, they must rely on documents provided by the police, which often lack descriptions of non-verbal behavior and emotions. Already in current practice, prosecutors struggle to evaluate information from written reports in order to make investigative and indictment decisions. Both in its current form and in potential GenAI systems, the written sources need to integrate more elaborate contextual information, verbal markers such as pitch, hesitation, and emphases, indicating emotional information (Bergman Blix, 2022), to allow for an accurate understanding and assessment of the case. It is also worthwhile to note that this example contradicts the common conception that emotions should be taken out of legal stories to secure correct information.

The problem of lacking emotional-interactional knowledge also applies to judges. Below, tribunal judge Lina (55+) develops on her methods to include not only verbal markers, but also body language as vital pieces of information in the transcriptions from a hearing:

*Judge Lina: Another thing that I do, that you might have noticed, is keeping track of aspects connected to non-verbal language, bodily communication. […] When people stop, cannot talk, are particularly emotional…I keep track of this in the minute of the hearing, but not by saying—“let us acknowledge that the woman is having an emotional moment,” because I do not want the person to feel unease, as if she’s under a sort of …examination. I say “do you want some water,” “I can see that you are not able to speak fluently, do you want to have a break,” “I can see that your moved, why?” So, this is something that it’s necessary to me both to get in contact with the witness and make her feeling that she’s not only a voice on the tape recorder, but a person listened to by another person….and to have a reflection of these events in the minutes. So when I read it, and I write something about the person in the motivation of the judgment, I can describe certain behaviors symptomatic of this … And this serves to the appeal. Because, if the judge of first instance says “it could be seen that she was emotional” but this does not have a validation in the minute, it’s more an interpretative truth, lacking a validation…I mean, you have to trust your colleague who felt that the person was struggling.*

In this first excerpt, Lina explains that she intervenes when witnesses struggle during the trial to put them at ease. Her use of professionally accepted cues (“do you want some water,” “do you want to have a break”) abide by the “limited repertoire” of judges to show empathy for witnesses without risking their impartial display (Bergman Blix and Wettergren, 2019). Moreover, these interventions are necessary to register in the minute the type of emotional reactions occurring in the courtroom, offering a validation of judge Lina’s interpretation of the person giving testimony both to the public, the parties and to the appeal court. Continuing on this line of reasoning, Lina offers an example from a recent case of IPV and rape of a young woman:

*Judge Lina: For example, when there was the little girl, the 17 years old little girl. She was really struggling, truly struggling. […] When she left, I said “let us acknowledge that the witness did these gestures [speaks in a very fast speed]: of touching her hair, of touching her neck, of stopping, of getting emotional, of not being able to speak, of looking for the therapist’s hand [slower speed]. I said these things, because for me it’s very important…that in the minutes there is track of how things happened, and I say that in the moment when things happen, before everyone. Because it’s not my interpretation, and if someone wants to contest the way I am summarizing the witness’ behavior, they can do that. Then, when the appellate judge read the minutes with those things and no one had contested this information, the appeal judge already has a support which is not the judge’s sensation, but what emerged during the trial.*

In this extract, judge Lina demonstrates the importance of reporting bodily and emotional communication in a way that is coherent with the orality, immediacy and adversarial principles. The orality and immediacy principles demand evidential information to be constructed in the presence of all involved parties in the courtroom (“*when* things happen, before *everyone*”). The adversarial principle allows for all parties to present and respond to arguments (“if someone wants to contest the way I am summarizing the witness’ behavior, they can do that”). We can also note that judge Lina’s interventions and descriptions imply an empathic attuning and understanding of the witness’ situation in court. On a substantial level, these pieces of bodily and emotional information are necessary to support Lina’s credibility assessments as outlined in the final judgment. In the Italian system, where the court of appeal evaluates evidence based on transcripts, these rich and nuanced texts allow for the appeal judges to understand and reassess the reasoning of the lower court. If predictive systems or GenAI should function in a legally sound way, they need to tag these types of information into annotations and develop methods to achieve valid interpretations.

## 5 Concluding discussion

Real judicial proceedings entail establishing the events at the center of the dispute, and interpreting and evaluating these events from a legal perspective. All these activities reduce the complexity of stories to fit within legal categories. The “skeletonization of facts so as to narrow moral issues to the point where determinate rules can be employed to decide them” is considered by Geertz (1983, p. 170) as the defining feature of the legal process. Nevertheless, it is vital that the reduction assists rather than hinders decision-making also from a procedural justice perspective (Remolina and Osa, 2024). Our illustrations show that legal professionals’ emotive-cognitive efforts aim at arriving at a reduction that is correct under the legal framework and has a hold on social reality. These efforts are evident both when prosecutors conduct the investigations and when judges deliberate, and are connected to the need of achieving the required level of certainty about the decision. Legal professionals try to make sense of the nuances of the case, using empathy and emotional-interactional information, to scrutinize and/or validate their interpretations of observed behaviors, in critical dialogue with the jurisprudence and the law (as shown in the Mimosa case with judge Ines).

In the everyday work at prosecution offices and courts, information gathering and transferring are produced in texts of different kinds, such as police reports, indictments, minutes, transcriptions, and judgments. Together, these texts compose the case file, which realizes a significant cut off of the full experiences of the trial, with its emotive-cognitive processes. Since “*quod non est in actis non est in mundo*” (what is not collected in the case file does not exist for case adjudication), the contextual information, as well as verbal, emotional, and bodily nuances and reactions not captured by the case file get lost (as demonstrated in prosecutor Stefano’s failure to decide on measures based on the police report in a IPV case). Legal professionals can try to remedy the loss of vital information by inventing their own methods for including these data into the case file, as illustrated by judge Lina. Nevertheless, the final text file, that is the judgment, in our material, always cuts off these types of behavioral and social information. This loss of information became clear when we compared the content of the deliberation with the written judgment about the same case (judges Enrico, Beatrice and Sonia in a IPV and rape case), noticing that the empathic attuning performed by the judges disappeared between the lines of the motivation. An Italian judgment is composed of various sections, explaining and linking facts with the reasons for the decision and the relevant laws. It follows that the judgment, while being the apex of the entire proceedings, captures a minimal amount of what happened from filing to disposition and during the deliberation.

So, in light of the importance of progressively purified texts in legal proceedings (Abbott, 1981), what can be a legitimate usage of GenAI, if any? As envisaged by the Courts and Tribunal service of England and Wales (Courts and Tribunals Judiciary, 2023), Gen AI can contribute to the skeletonization of the full trial experience by summarizing the content of the case file, for example by abridging the transcripts of the hearings, the procedural documents filed by prosecutor and defense, or the experts’ reports. To some extent, this function is in continuity with the skeletonization work done by judges. However, since the capacity of these models to capture what matters from a factual, legal perspective is rooted on statistical analysis and not on actual legal practice, the quality of their outputs cannot be taken for granted and must be verified on a case-by-case basis. Judges can ask GenAI to do the job of summarizing documents of the case file, but they need to confront the output with their full knowledge of the document summarized and of the events described. Using the summary without verifying its content open the door to potential bias and removal of key pieces of information. If adequately checked and implemented with the emotional-interactional information collected during trial, GenAI’s summaries can positively assist judges and prosecutors in their work. The risk, however, is the viability of said quality check, as caseload pressure can push judges to focus on summaries without controlling their quality.

Predictive systems, as described earlier, base their predictions on previous judgments that are radically simplified expositions of facts, legal issues and their connections. As argued here, emotions, non-verbal behaviors and the nuances of the case are cut off in the judgments. The prediction is thus based on a subset of the data generated by previous trials, but with several blind spots about components that have a relevant role in the decision. Differently from GenAI predictive systems work through a process of digital codification of the text into annotations and relations requiring human supervision from persons with legal expertise, at least in the form discussed in this paper (Galli and Sartor, 2023, pp. 173–4).

Another potential challenge is predictive systems’ timing in selecting and simplifying the nuances and richness of the proceeding, the history of those involved, and several pieces of information that judges, as shown in our analysis, normally consider. Predictive systems imply a jump to the conclusions of the case. As shown in our first example where judge Ines came back to nuanced details in her dialogue with jurisprudence in the late stages of the deliberation, these queries, if made to a predictive system, could not have been answered, since what was cut off during the simplification discussed above cannot be regenerated. Furthermore, if these details are cut off in the simplification process of the predictive system, two cases can seem identical, although they carry important distinctive elements. This is particularly problematic since the logic of predictive systems conceals all the details not captured by semantic annotations. Lastly, the more judges and prosecutors are pressed by caseload and performance expectations, the more they will be tempted to rely on GenAI summaries and predictive devices, losing effective human control and putting high demands on correct machine-made justice.

## Data Availability

The datasets presented in this article are not readily available because confidentiality due to cases dealing with criminal proceedings. Requests to access the datasets should be directed to stina.bergmanblix@uu.se.

## Footnotes

^1^The Act is the first comprehensive regulation of AI, establishing Regulation (EU) 2024/1689 of the European Parliament and of the Council of 13 June 2024 laying down harmonized rules on artificial intelligence and amending Regulations (EC) No 300/2008, (EU) No 167/2013, (EU) No 168/2013, (EU) 2018/858, (EU) 2018/1139 and (EU) 2019/2144 and Directives 2014/90/EU, (EU) 2016/797 and (EU) 2020/1828 (Artificial Intelligence Act) (Text with EEA relevance) requirements and duties based on the risk of causing harm by products and services building on AI systems. The act classifies four categories of risk (“unacceptable,” “high,” “limited” and “minimal”), and one additional category for general-purpose AI.

^2^Recidivism risk assessment builds on decision support systems designed to suggest precautionary measures like pre-trial detention or the final sentence. Since these kinds of decisions are not the focus of our study, they will not be included in the discussion.

^3^Unstructured simply means that data are not organized into structured database formats. Unstructured data has an internal structure, but it is not predefined through data models.

^4^In some cases, judgments are supplemented by other case files’ documents.

^5^The system was developed by the Court of Appeal in cooperation with the Law Department and the Department of Information Engineering of the local University. See https://giustiziapredittiva.unibs.it/ [Last visited August 11, 2023].

^6^See also the project website at https://www.predictivejurisprudence.eu/.

^7^This is the promise of LISIA, a legal tech offering natural language search on a large jurisprudential database https://Lisia.it.

^8^A fuller presentation goes beyond the scope of this paper.

^9^Taking a broader perspective, other scopes emerge. For instance, considering case parties, the scope of the proceeding and of the trial is to assure procedural justice, hence, to show that justice is done.
